# SARS-CoV-2 Prevalence in Malawi Based on Data from Survey of Communities and Health Workers in 5 High-Burden Districts, October 2020

**DOI:** 10.3201/eid2813.212348

**Published:** 2022-12

**Authors:** Joe Alex Theu, Alinune Nathanael Kabaghe, George Bello, Evelyn Chitsa-Banda, Matthews Kagoli, Andrew Auld, Jonathan Mkungudza, Gabrielle O’Malley, Fred Fredrick Bangara, Elizabeth F. Peacocke, Yusuf Babaye, Wingston Ng’ambi, Christel Saussier, Ellen MacLachlan, Gertrude Chapotera, Mphatso Dennis Phiri, Evelyn Kim, Mabvuto Chiwaula, Danielle Payne, Nellie Wadonda-Kabondo, Annie Chauma-Mwale, Titus Henry Divala, Public Health Institute of Malawi COVID-19 surveillance committee

**Affiliations:** University of Washington Malawi International Training and Education Center for Health, Lilongwe, Malawi (J.A. Theu, G. Bello, J. Mkungudza, F.F. Bangara, Y. Babaye, C. Saussier);; US Centers for Disease Control and Prevention, Malawi, Lilongwe (A.N. Kabaghe, A. Auld, E. Kim, D. Payne, N. Wadonda-Kabondo);; Public Health Institute of Malawi, Lilongwe (E. Chitsa-Banda, M. Kagoli, M. Chiwaula, A. Chauma-Mwale);; University of Washington International Training and Education Center for Health, Seattle, Washington, USA (G. O’Malley, E. MacLachlan);; Norwegian Institute of Public Health, Lilongwe (E.F. Peacocke);; Kamuzu University of Health Sciences, Blantyre, Malawi (G. Chapotera, M.D. Phiri, T.H. Divala)

**Keywords:** SARS-CoV-2, COVID-19, community health workers, community surveillance, serosurveillance, respiratory infections, viruses, Malawi, severe acute respiratory syndrome coronavirus 2, coronavirus disease, zoonoses

## Abstract

To determine early COVID-19 burden in Malawi, we conducted a multistage cluster survey in 5 districts. During October–December 2020, we recruited 5,010 community members (median age 32 years, interquartile range 21–43 years) and 1,021 health facility staff (HFS) (median age 35 years, interquartile range 28–43 years). Real-time PCR–confirmed SARS-CoV-2 infection prevalence was 0.3% (95% CI 0.2%–0.5%) among community and 0.5% (95% CI 0.1%–1.2%) among HFS participants; seroprevalence was 7.8% (95% CI 6.3%–9.6%) among community and 9.7% (95% CI 6.4%–14.5%) among HFS participants. Most seropositive community (84.7%) and HFS (76.0%) participants were asymptomatic. Seroprevalence was higher among urban community (12.6% vs. 3.1%) and HFS (14.5% vs. 7.4%) than among rural community participants. Cumulative infection findings 113-fold higher from this survey than national statistics (486,771 vs. 4,319) and predominantly asymptomatic infections highlight a need to identify alternative surveillance approaches and predictors of severe disease to inform national response.

The first 3 SARS-CoV-2 infections in Malawi were confirmed on April 2, 2020, using real-time PCR (rPCR) (*1*). Facility-based national surveillance data and national statistics indicated that the number of new infections with SARS-CoV-2, the virus that causes COVID-19, rose rapidly in June 2020 and peaked in mid-July at 192 cases/day before declining to a 7-day moving average of 2–6 cases/day in October 2020 (Appendix). Daily test average positivity declined from 17.5% in July to 2.7% by October 2020.

The national COVID-19 surveillance and response in Malawi, like those of most public health systems in Africa, relies on routine facility-based surveillance data sent from district and regional health offices, which presents several challenges. First, without a reliable denominator for estimating key epidemiologic parameters, the source population is poorly defined. Second, a substantial proportion of the infected population who are asymptomatic or mildly ill might not seek treatment at health facilities and might thus remain undetected (*2*–*4*). Third, because of low availability of reagents and low investment in the healthcare system, low capacity for SARS-CoV-2 testing limits diagnosis (*5*). In addition, some community members might avoid COVID-19 tests because of negative perceptions about the disease or healthcare system (*6*).

Apart from information from small surveys in urban areas (M.B. Chibwana, unpub. data, https://www.medrxiv.org/content/10.1101/2020.07.30.20164970v3), the extent of COVID-19 spread and associated demographic and clinical characteristics has remained undescribed in Malawi, making it difficult to interpret morbidity and mortality data and obstructing evidence-informed predictive modeling and planning. We therefore conducted a healthcare facility and population-based survey to determine viral and antibody prevalence and risk factors for SARS-CoV-2 infection in 5 districts of Malawi.

## Methods

### Study Design and Study Population

**Figure 1:**
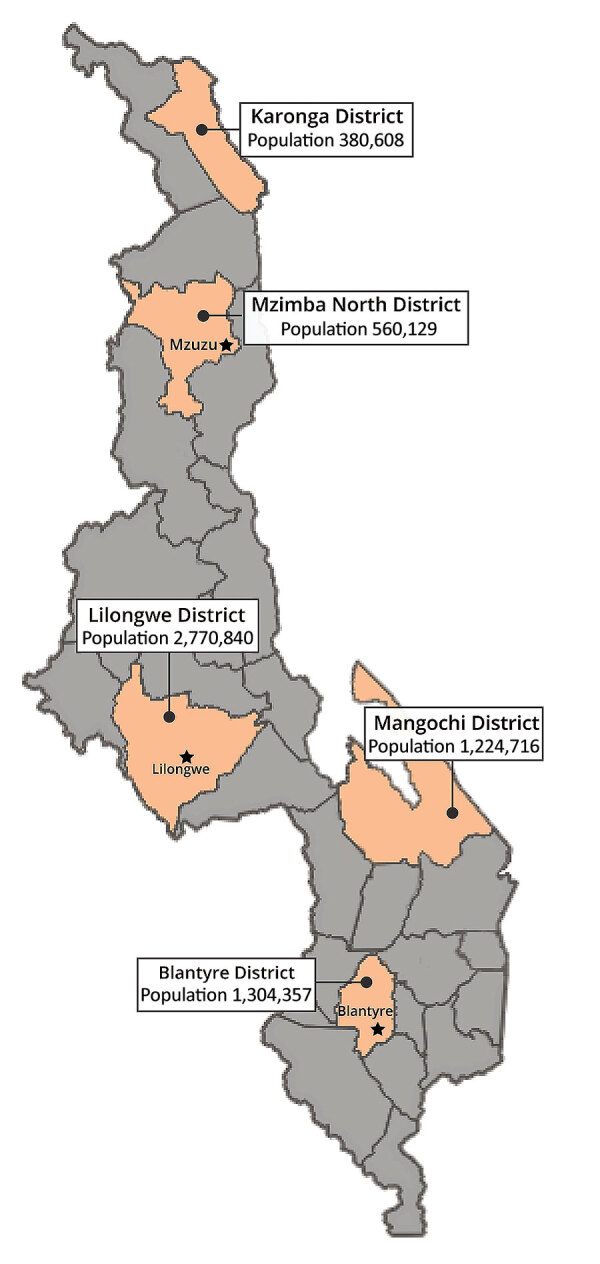
Locations and populations of districts included in study of SARS-CoV-2 infection in Malawi, 2020.

During October 14–December 8, 2020, we conducted a cross-sectional survey in 3 districts with urban centers (Lilongwe, Blantyre, and Mzimba North) and in 2 predominantly rural districts (Karonga and Mangochi) (Figure 1) from among the 28 districts in Malawi. The 5 districts selected for the survey were categorized as high-risk areas for SARS-CoV-2 infections because of high population density, high volume of travelers to and from high-risk countries, or both. At the beginning of the survey, Lilongwe district had reported 49 cases/100,000 population, Blantyre 151/100,000 population, Mzimba North 101/100,000 population, Karonga 22/100,000, and Mangochi 12/100,000 population (Appendix).

The survey population was composed of community members >10 years of age and health facility staff (HFS) >18 years of age. Participants >18 years of age provided written consent to be included in the survey; participants <18 years of age provided personal assent and consent from a guardian. All HFS—frontline healthcare workers and support and administrative staff from primary, secondary, and tertiary facilities—were eligible for the survey if they consented.

### Sample Size and Sampling Method

The target sample size for community participants from each district was <1,620 from 540 households, <8,100 participants from 2,700 households overall. We based sample size targets on several assumptions about general population participants: 6% of the surveyed population would test rPCR positive on the basis of a rPCR positivity rate from national surveillance data of 6%–6.5% in early to mid-June 2020 (Appendix); +10% precision for the 95% CI for the rPCR-confirmed SARS-CoV-2 infection prevalence; an arbitrary design effect of 1.3; response rate of 96%; and 1% of sampled households with fewer than the targeted number of participants. For HFS, the total sample size was 1,600 assuming rPCR-confirmed SARS-CoV-2 infection prevalence of 12% (*7*), +15% precision for the 95% CI, an arbitrary design effect of 1.2, and expected response rate of 95%.

For community participants, we used a 3-stage cluster sampling approach to randomly select 27 (16 rural and 11 urban) enumeration areas (EAs) using probability proportional to size of EA in each district. Four sampled EAs were noncooperative because of misconceptions about COVID-19 and were replaced by reserve EAs also randomly selected using probability proportional to size. From the selected EAs, we used a simple random sampling approach using random number tables to sample 20 households per EA from the 2018 national census household listing obtained from the Malawi National Statistics Office. We entered names and ages of all household members to an electronic tablet using an OpenDataKit (ODK; https://getodk.org) mobile application. Using a command programmed in the ODK form in the tablet, we randomly selected a maximum of 3 names from among household members >10 years of age to participate. For households with <3 household members >10 years of age, we selected all age-eligible members to participate.

We included 40 facilities for the HFS survey. In each district, we first selected the largest facility, a secondary or tertiary hospital, to maximize the number of included HFS, then used probability proportional to size sampling for an additional 7 primary or secondary care facilities in each district (Appendix). We used the same approach to list and sample HFS using the ODK program command to select 400 HFS per district in Blantyre, Lilongwe, and Mzimba North and 200 per district from Karonga and Mangochi. We sampled more HFS from facilities in urban than predominantly rural districts because they have more staff. In facilities where the number of HFS was less than or equal to the target sample size, we included all staff.

### Community Sensitization and Data Collection

A trained survey team met with community leaders including district commissioners, district councilors, chiefs, and subchiefs. Community members were mobilized through meetings coordinated with village navigators, community health workers, and the survey team. Public address systems were used to transmit messages about the survey to the community. At health facilities, we briefed the district health officer and participating health facility managers before they conducted sensitization meetings with HFS.

Study staff equipped with required personal protective equipment visited sampled households and health facilities to obtain informed consent and enroll participants. We collected data using an electronic questionnaire on an ODK platform and sent them to a server hosted at the Malawi Central Health Surveillance Unit. We collected information on sociodemographics, international travel, gatherings attended, contact with rPCR-confirmed SARS-CoV-2–infected persons, self-reported underlying health conditions, and signs and symptoms of influenza-like illness or severe acute respiratory illness in the previous 6 months.

### Laboratory Procedures

We collected nasopharyngeal swabs and blood specimens and transported them to testing laboratories under cold chain processes and stored them in cryovials in a −80°C freezer until they were analyzed. Nasopharyngeal specimens were tested in government laboratories for SARS-CoV-2 RNA using rPCR for the RdRp (RNA-dependent RNA polymerase) and N (nucleocapsid) genes using the Abbott RealTime SARS-CoV-2 Assay (Abbott Molecular Inc., https://www.molecular.abbott). Serum specimens were analyzed using the Wantai SARS-CoV-2 Ab ELISA (https://www.fda.gov/media/140030/download) for qualitative detection of total antibodies (IgG and IgM) to SARS-CoV-2, a 2-step incubation antigen sandwich enzyme immunoassay kit using polystyrene microwell strips precoated with recombinant SARS-CoV-2 receptor-binding domain (RBD) antigen. The manufacturer-reported performance characteristics for the Wantai test were 96.7% (95% CI: 83.3%–99.4%) sensitivity and 97.5% (CI: 91.3%–99.3%) specificity. We calculated the ratio between absorbance and cutoff points for each specimen; ratios <0.9 indicated specimens were SARS-CoV-2–negative, ratios >1.1 positive, and ratios 0.9–1.1 borderline. All specimens with initial positive or borderline results were retested using the same assay before final determination of status. If initial and retest results did not match, we used a EUROIMMUN SARS-CoV-2 IgA and IgG assay test kit (https://www.euroimmun.com) for verification. 

### Data Analysis

The primary outcomes we used to define infection positivity were any positive test result for either SARS-CoV-2 RNA from an rPCR test or SARS-CoV-2 RBD total antibodies from the Wantai ELISA test. Other outcomes included self-reported influenza-like illness and severe acute respiratory illness signs and symptoms for those with a positive primary outcome. Independent variables in the analysis included age, sex, location, highest level of education, occupation, self-reported underlying medical conditions, and reported high risk for contact with SARS-CoV-2. We performed all statistical analyses using Stata software version 14.1 (https://www.stata.com). We calculated sampling weights for community participants on the basis of the 2018 Malawi population and housing census (*7*) and for HFS, on the basis of the 2019 Malawi Harmonized Health Facility Assessment (*8*). We used Svy commands in Stata to calculate proportions to account for the complex survey design and incorporate sampling weights to address unequal selection probability within districts. We calculated SARS-CoV-2 infection prevalence with 95% CIs. We used adjusted seroprevalence results to estimate the number of SARS-CoV-2 infections in the 5 districts. We used bivariate logistic regression analysis to calculate crude odds ratios (ORs) and multivariable logistic regression analysis to calculate adjusted odds ratios (aORs) with 95% CIs. In the multivariable analysis, we included age and sex and variables statistically significant at p<0.05 during bivariate regression.

The National Health Sciences Research Committee (NHSRC) in Malawi, as the engaged institution, reviewed and approved the protocol. The US Centers for Disease Control and University of Washington provided a nonresearch determination under Code of Federal Regulations, Common Rule (45 CFR 46.102(l) (*2*). Sampled persons provided verbal consent or assent to participate after understanding the purpose, procedures, risks and benefits of the study. We ensured that data were collected in a private area and electronic data access was password-controlled.

## Results

### Participant Recruitment and Data Collection

**Figure 2:**
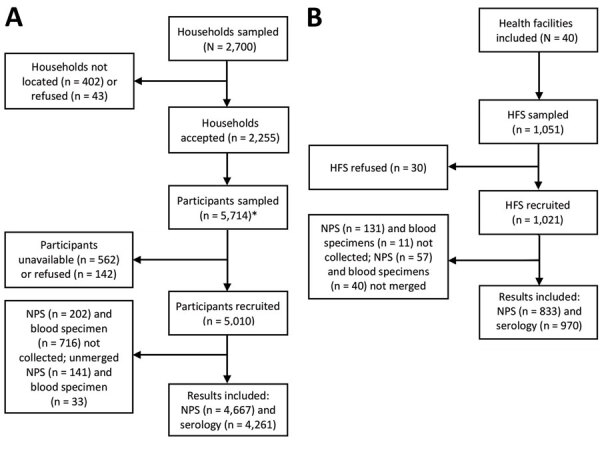
Flowchart for study of SARS-CoV-2 infection in Malawi, 2020, showing participants included and lost to follow-up among household residents and health facility staff initially sampled. A) Among the 2,255 community households accepted into the study, 17.8% had 1 eligible participant, 25.8% had 2, and 56.4% had 3. B) The 1,051 HFS initially sampled were recruited from 40 health facilities. HFS, health facility staff; NPS, nasopharyngeal specimen.

We chose 2,700 households to sample, from which we did not locate 402 (14.9%) and 43 (1.6%) refused to participate (Figure 2, panel A). Among the 2,255 households that consented, 983 had <3 eligible persons in the household. Overall, we sampled 5,714 household members and enrolled 5,010 (87.7%). Among the community participants enrolled, 4,667/5,010 provided nasopharyngeal and 4,261/5,010 blood specimens with results available for analysis. For HFS, we sampled 1,051 and enrolled 1,021 (97.1%) (Figure 2, panel B). Among samples taken from enrolled participants, 833/1,021 provided nasopharyngeal and 970/1,021 blood specimens with results available for analysis.

### Participant Characteristics

Weighted proportions of 63.4% of community participants and 52.5% of HFS were women (Table 1). Median age was 32 years (interquartile range 21–43 years) among community participants and 35 years (interquartile range 28–4 years) among HFS. Among community participants, 53.3% had primary and 29.0% had secondary education; among HFS, most of them nurses, 58.9% had secondary education and 36.5% had tertiary education (Appendix). Overall, 46.0% of community participants reported being unemployed. The largest proportion of both community and HFS participants were from Mzimba North. Among community participants 49.5% and among HFS 64.7% were from urban settings. An underlying medical condition was reported by 23.9% of HFS and 11.2% of community participants.

**Table 1:** Characteristics of participants in survey of SARS-CoV-2 prevalence in Malawi, October 2020*

Characteristic	Community participants, n = 4,261			Health facility staff, N = 970		Total, N = 5,231
	No. (%)	Weighted proportion (95% CI)		No. (%)	Weighted proportion (95% CI)	
Sex						
M	1,524 (35.8)	36.6 (32.8–40.6)		428 (44.1)	47.5 (39–56)	1,952
F	2,737 (64.2)	63.4 (59.4–67.2)		542 (55.9)	52.5 (44–61)	3,279
Age, y						
10–19	982 (23.0)	20.1 (18.0–22.4)		8 (0.8)	0.4 (0.1–1.5)	990
20–29	1,085 (25.5)	23.8 (21.9–25.8)		291 (30.0)	20.0 (14.8–26.3)	1,376
30–39	887 (20.8)	21.7 (19.4–24.3)		335 (34.5)	38.7 (33.1–44.6)	1,222
40–49	626 (14.7)	16.8 (15.1–18.7)		236 (24.3)	25.9 (22.6–29.5)	862
≥50	681 (16)	17.5 (15.5–19.8)		100 (10.3)	15.0 (9.6–22.6)	781
District						
Blantyre	535 (12.6)	16.0 (13.0–19.6)		163 (16.8)	15.1 (7.9–27.0)	698
Karonga	1,092 (25.6)	8.5 (7.2–10.1)		132 (13.6)	19.9 (11.5–32.1)	1,224
Lilongwe	560 (13.1)	37.8 (29.9–46.5)		216 (22.3)	23.6 (17.8–30.5)	776
Mangochi	937 (22.0)	23.9 (19.8–28.6)		191 (19.7)	9.5 (7.2–12.4)	1,128
Mzimba North	1,137 (26.7)	13.7 (11.4–16.4)		268 (27.6)	31.9 (22.6–42.9)	1,405
Location type						
Rural	1,505 (35.3)	50.5 (38.3–62.5)		406 (41.9)	35.2 (32.1–38.2)	1,911
Urban	2,756 (64.7)	49.5 (37.5–61.6)		564 (58.1)	64.7 (61.6–67.7)	3,320
Household size, categorical						
1–2	500 (11.7)	15.1 (11.5–19.6)		241 (24.8)	23.5 (17.7–30.5)	741
3–4	1,888 (44.3)	44.1 (41.1–47.1)		331 (34.1)	34.5 (29.4–40.1)	2,219
≥5	1,872 (43.9)	40.8 (36.9–44.8)		398 (41.0)	42.0 (36.0–48.1)	2,270
Education†						
No education	339 (8.0)	12.8 (8.8–18.2)		0	0	339
Primary	2,138 (50.5)	53.3 (48.0–58.5)		51 (5.3)	4.6 (2.5–8.4)	2,189
Secondary	15,250 (35.9)	29.0 (25.0–33.4)		485 (50.0)	58.9 (51.5–66.0)	2,005
Tertiary/postsecondary	237 (5.6)	4.9 (3.7–6.5)		434 (44.7)	36.5 (29.3–44.3)	671
Occupation						
Student	950 (22.3)	18.5 (16.4–20.8)		NA	NA	950
Unemployed	1,704 (40.0)	46.0 (40.8–51.2)		NA	NA	1,704
Employed, HFS	30 (0.7)	0.98 (0.7–1.4)		970	970	65
Employed, non-HFS	275 (6.5)	0.54 (0.3–0.9)		NA	NA	30
Retired	65 (1.5)	7.0 (5.4–9.1)		NA	NA	275
Other	1,237 (29.0)	27.0 (22.6–31.9)		NA	NA	1,237
Preexisting medical conditions						
Any medical condition	472 (11.1)	11.2 (9.6–13.0)		175 (18.0)	23.9 (19.6–28.9)	647
Diabetes mellitus	38 (0.9)	0.7 (0.4–1.1)		11 (1.1)	0.7 (0.3–1.6)	49
CVD, including hypertension	224 (5.3)	5.5 (4.3–6.9)		68 (7.0)	10.5 (6.3–16.9)	292
Renal disease	2 (0)	0.04 (0.01–0.21)		4 (0.4	1.0 (0.3–3.2)	6
Immunosuppressive condition‡	78 (1.9)	1.8 (1.3–2.5)		39 (4.0%)	5.7 (2.6–11.8)	117
Obesity	12 (0.3)	0.2 (0.1–0.4)		10 (1.0)	0.6 (0.2–1.4)	22
Asthma	104 (2.5)	2.4 (1.8–3.2)		47 (4.8)	6.9 (4.4–10.4)	151
Chronic lung disease, including COPD	8 (0.2)	0.08 (0.04–0.2)		2 (0.2)	0.1 (0.03–0.5)	10
Liver disease	3 (0.1)	0.05 (0.01–0.20)		2 (0.2)	1.0 (0.15–6.0)	5
Other disease	65 (1.6)	2.1 (1.5–2.9)		11 (1.1)	1.4 (0.46–4.2)	76

*COPD, chronic obstructive pulmonary disease; CVD, cardiovascular disease; HFS, health facility staff; NA, not applicable. †The highest level of education attained. Primary education = 8 y; secondary education = 4 y; tertiary/postsecondary = college/university education. ‡From cancer, chemotherapy, radiation therapy, immunosuppressive medications, self-reported HIV, organ transplant, or inherited immunodeficiency.

### Prevalence of rPCR-Confirmed SARS-CoV-2 Infection

Of 4,667 specimens collected from community participants that were tested for SARS-CoV-2 by rPCR, 14 (0.3%, 95% CI 0.2%–0.5%) were positive. The prevalence was highest among community participants ≥50 years of age (0.5%, 95% CI 0.1%–1.3%). No rPCR-confirmed SARS-CoV-2 infection was observed in participants 10–14 years of age. Of the 851 specimens collected from HFS, 4 (0.5%, 95% CI 0.1%–1.2%) tested positive. Prevalence was highest among participants 30–49 years of age (0.8%, 95% CI 0.2%–2.0%) and significantly higher among male participants (1.0%, 95% CI 0.3%–2.6%) than among female participants (0.0%, 95% CI 0.0%–0.8%) (p = 0.004).

### Seroprevalence of SARS-CoV-2 RBD Total Antibodies

Overall SARS-CoV-2 seroprevalence among community participants was 7.8% (95% CI 6.3%–9.6%) and similar between male participants (8.3%, 95% CI 6.5%–10.4%) and female participants (7.5%, 95% CI 6.0%–9.4%) (Table 2). Participants 30–39 and ≥50 years of age had higher seroprevalence than did other age groups. Seroprevalence was highest in Blantyre (13.1%; 95% CI 9.0%–18.7%) and Mzimba North (12.1%, 95% CI 8.7%–16.6%) and lowest in Mangochi (4.1%, 95% CI 2.6%–6.2%). Overall, the seroprevalence was higher in urban (12.6%, 95% CI 11.2%–14.1%) than rural areas (3.1%, 95% CI 1.8%–5.5%). SARS-CoV-2 seroprevalence among HFS was 9.7% (95% CI 6.4%–14.5%). Seroprevalence was similar by sex; there was a nonsignificant 2-fold difference in seroprevalence between participants in urban (14.5%, 95% CI 9.7%–21.1%) and rural (7.4%, 95% CI 3.6%–14.7%) locations.

**Table 2:** Prevalence of and risk factors associated with SARS-CoV-2 positive test result among participants in survey of SARS-CoV-2 prevalence in Malawi in October 2020*

Characteristic	Community, N = 4,261					Health facility staff, N = 970			
	Pos	Weighted prevalence (95% CI)	Crude OR (95% CI)	Adjusted OR (95% CI)†		Pos	Weighted prevalence (95% CI)	Crude OR (95% CI)	Adjusted OR (95% CI)†
Overall	423	7.8 (6.3–9.6)	NA	NA		124	9.7 (6.4–14.5)	NA	NA
Sex									
M	170	8.3 (6.5–10.4)	Referent	Referent		58	9.7 (5.6–15.6)	Referent	Referent
F	253	7.5 (6.0–9.4)	0.9 (0.7–1.1)	0.9 (0.6–1.2)		66	9.7 (6.1–15.1)	1.0 (0.6–1.6)	0.7 (0.4–1.0)
Age, y									
10–19	71	5.3 (3.7–7.5)	Referent	Ref		5	57.4 (4.4–97.5)	Ref	Referent
20–29	104	6.9 (5.3–9.0)	1.3 (0.9–1.9)	1.3 (0.8–2.4)		38	7.6 (4.4–12.9)	0.06 (0.01–0.3)§	0.1 (0.01–0.5)¶
30–39	105	9.7 (7.4–12.6)	1.9 (1.3–3.0)§	2.6 (1.3–5.2)§		41	12.6 (6.4–23.4)	0.1 (0.02–0.4)§	0.2 (0.03–0.9)¶
40–49	61	6.2 (4.0–9.5)	1.2 (0.8–1.8)	1.8 (0.9–3.6)		32	6.6 (3.3–13.0)	0.1 (0.01–0.3)§	0.1 (0.01–0.7)¶
≥50	82	11.0 (7.6–15.8)	2.2 (1.4–3.4)¶	2.8 (1.4–5.4)§		8	8.7 (2.6–25.4)	0.1 (0.01–0.4)§	0.2 (0.02–1.2)
District									
Karonga	103	9.4 (7.3–12.2)	Referent	Referent		13	8.9 (2.2–29.4)	Referent	Referent
Blantyre	67	13.1 (9.0–18.7)	1.4 (0.9–2.2)	1.3 (0.9–2.1)		25	4.7 (1.1–18.0)	3.5 (0.8–15.2)	3.6 (0.6–21.4)
Lilongwe	47	6.1 (3.5–10.2)	0.6 (0.3–1.1)	0.9 (0.6–1.6)		23	10 (6.7–14.7)	2.3 (0.6–8.6)	2.1 (0.4–10.2)
Mangochi	38	4.1 (2.6–6.2)	0.4 (0.2–0.6)§	0.5 (0.3–0.8)§		6	2.6 (1.0–6.6)	0.5 (0.1–2.5)	0.7 (0.1–3.5)
Mzimba North	168	12.1 (8.7–16.6)	1.3 (0.8–2.1)	1.5 (1.0–2.2)		57	14.5 (6.7–28.5)	2.0 (0.3–12.0)	2.0 (0.4–11.3)
Location type									
Rural	59	3.1 (1.8–5.5)	Referent	Referent		27	7.4 (3.6–14.7)	Referent	Referent
Urban	364	12.6 (11.2–14.1)	4.4 (2.5–7.9)¶	4.2 (2.6–6.7)¶		97	14.5 (9.7–21.1)	2.1 (0.9–4.9)	1.8 (0.7–4.9)
Household size									
1–2	47	8.4 (5.9–11.8)	Referent	–		33	8.3 (3.6–17.9)	Referent	NA
3–4	167	7.4 (5.7–9.4)	0.9 (0.6–1.3)	NA		35	8.3 (3.9–16.9)	1.0 (0.3–3.9)	NA
≥5	209	8.0 (6.1–10.4)	1.0 (0.6–1.5)	NA		56	11.6 (7.3–17.9)	1.4 (0.6–3.5)	NA
Education#									
No education	22	7.6 (4.2–13.2)	Referent	Referent			NA	NA	NA
Primary	151	5.2 (3.8–7.1)	0.7 (0.4–1.3)	0.4 (0.2–0.9)¶		3	5.1 (1.7–14.3)	Referent	NA
Secondary	211	11.9 (9.7–14.4)	1.6 (0.9–3.1)	0.8 (0.4–1.7)		53	8.6 (5.6–13.0)	1.7 (0.6–5.5)	NA
Tertiary/postsecondary	39	14.4 (10.6–19.3)	2.1 (1.0–4.1)¶	0.8 (0.4–1.8)		68	12.0 (7.1–19.7)	2.5 (0.7–8.9)	NA
Occupation									
Student	81	6.7 (4.6–9.8)	Referent	Referent			NA	NA	NA
Unemployed	160	7.7 (6.1–9.7)	1.2 (0.8–1.7)	0.7 (0.4–1.4)			NA	NA	NA
Employed, HFS	13	4.9 (1.1–18.6)	0.7 (0.2.3.3)	0.2 (0.05–1.1)			NA	NA	NA
Employed, non-HFS	3	10.0 (6.4–15.2)	1.5 (0.9–2.7)	0.6 (0.2–1.2)			NA	NA	NA
Retired	36	25.7 (14.5–41.3)	4.8 (2.1–10.9)¶	1.4 (0.5–4.0)			NA	NA	NA
Other	130	7.6 (5.5–10.4)	1.1 (0.7–1.8)	0.6 (0.3–1.2)			NA	NA	NA
Contact with confirmed case									
No	422	7.8 (6.3–9.6)	Referent	NA		117	9.5 (6.1–14.4)	Referent	NA
Yes	1	14.5 (0.14–95.3)	2.0 (0.3–14.9)	NA		7	26.1 (8.5–57.3)	3.4 (0.9–12.7)	NA
Attended gatherings**									
No	90	6.9 (4.8–9.8)	Referent	NA		19	9.8 (6.3–15.0)	Referent	NA
Yes	333	8.3 (6.7–10.3)	1.2 (0.8–1.8)	NA		105	9.7 (6.0–15.3)	1.0 (0.5–2.0)	NA
Any medical condition									
No	367	7.7 (6.2–9.5)	Referent	NA		103	10.1 (6.8–14.7)	Referent	NA
Yes	56	8.7 (5.8–13.0)	1.1 (0.8–1.7)	NA		21	8.3 (3.3–19.4)	0.8 (0.3–2.0)	NA
Diabetes mellitus									
No	401	7.5 (6.0–9.3)	Referent	Referent		120	9.8 (6.2–15.3)	Referent	Referent
Yes	10	27.3 (12.9–48.9)	4.6 (1.7–12.5)§	2.4 (0.9–6.3)		2	17.6 (0.9–84.2)	2.0 (0.2–16.2)	1.9 (0.3–11.6)
CVD, including hypertension									
No	383	7.6 (6.1–9.4)	Referent	NA		113	10.6 (6.8–16.2)	Referent	NA
Yes	30	8.4 (4.6–14.9)	1.1 (0.6–2.0)	NA		9	3.3 (0.65–15.5)	0.3 (0.6–1.4)	NA
Immunosuppressive condition††									
No	398	7.5 (6.1–9.3)	Referent	NA		116	9.0 (5.7–13.9)	Referent	NA
Yes	9	12.0 (5.8–22.9)	1.7 (0.7–3.8)	NA		5	23.1 (8.1–50.7)	3.1 (1.7–8.7)¶	NA
Obesity									
No	407	7.6 (6.1–9.3)	Referent	NA		122	10.0 (6.3–15.5)	NA	NA
Yes	2	19.2 (3.3–6.2)	2.9 (0.5–15.9)	NA		0	0	NA	NA
Asthma									
No	407	7.6 (6.1–9.3)	Referent	NA		119	10.5 (6.6–16.3)	Referent	NA
Yes	2	9.5 (4.6–18.5)	1.3 (0.6–2.7)	NA		3	1.9 (0.34–9.7)	0.2 (0.03–0.8)¶	NA
Chronic lung disease, including COPD									NA
No	411	7.7 (6.2–9.4)	Referent	NA		121	9.9 (6.2–15.4)	Referent	NA
Yes	1	9.7 (0.05–69.6)	1.3 (0.1–12.2)	NA		1	59.1 (–)	13.1 (0.7–246.7)	NA
Pregnant									
No	88	7.6 (6.0–9.6)	Referent	NA		66	10.0 (6.3–15.7)	NA	NA
Yes	9	6.3 (2.8–13.4)	0.8 (0.3–1.9)	NA		0	0	NA	NA
Smoking									
No	400	7.9 (6.4–9.7)	Referent	NA		120	10.1 (6.6–15.1)	NA	NA
Yes, current	4	5.5 (0.9–26.5)	0.7 (0.1–3.7)	NA		0	0	NA	NA
Yes, past	19	6.0 (3.3–10.5)	0.7 (0.4–1.3)	NA		4	4.2 (0.61–24.3)	0.4 (0.05–2.9)	NA
International travel‡‡									
No	417	7.8 (6.3–9.6)	Referent	NA		13	9.6 (6.2–14.5)	Referent	NA
Yes	5	12.4 (3.0–39.8)	1.7 (0.4–6.5)	NA		3	18.0 (3.7–55.7)	2.1 (0.6–7.7)	NA

*COPD, chronic obstructive pulmonary disease; CVD, cardiovascular disease; HFS, health facility staff; NA, not available; OR, odds ratio; pos, positive. †Adjusted for age, sex, district and residence. ‡p value <0.05. §p value <0.01. ¶p value <0.001. #Education: primary education = 8 y; secondary education = 4 y; tertiary/postsecondary = college/university education. **Gathering included any of prayers, sporting events or market, family celebrations, weddings, funerals and bars. ††From cancer, chemotherapy, radiation therapy, immunosuppressive medications, self-reported HIV, organ transplant, or inherited immunodeficiency. ‡‡International travel includes both road and air travel.

We found significant association between community participants self-reporting diabetes and testing seropositive for SARS-CoV-2 in the crude data analysis (crude OR 4.6, 95% CI 1.7–12.5) but not in the adjusted analysis (aOR 2.4, 95% CI 0.9–6.3). Odds of testing seropositive for SARS-CoV-2 were higher among HFS reporting than those not reporting an immunosuppressive condition (aOR 3.1, 95% CI 1.7–8.7), but HFS reporting asthma were less likely to test positive (aOR 0.2, 95% CI 0.03–0.8). In the community participant survey, data on age, district, education, and location remained significant in the multivariable analysis (Table 2).

### Signs and Symptoms of SARS-CoV-2 Infection among Seropositive Participants

Among community participants who had a seropositive result, 84.7% reported having no COVID-19–associated signs or symptoms in the 6 months before the survey; 10.6% reported coughing, 9.2% runny nose, and 5.2% muscle pain (Table 3). One (0.7%) seropositive community participant reported being hospitalized, but admission details were unavailable. Among seropositive HFS participants, 76.0% reported no signs or symptoms, 16.6% runny nose, 6.8% fever, 3.6% sore throat, and 2.7% loss of smell; none were hospitalized.

**Table 3:** SARS-CoV-2 signs and symptoms in survey participants with a seropositive test result, Malawi, October 2020

Signs/symptoms of SARS-CoV-2 infection in previous 6 mo	Community participants, n = 423			Health facility staff, n = 124	
	No.*	Weighted % (95% CI)		No.*	Weighted % (95% CI
None	368	84.7 (78.4–89.4)		107	76.0 (57.9–87.9)
Fever	12	3.5 (1.7–6.8)		6	6.8 (2.6–17.7)
Shortness of breath	2	0.6 (0.11–3.2)		1	1.1 (0.14–7.9)
Sore throat	3	0.8 (0.2–2.7)		4	3.6 (1.2–10.2)
Runny nose	27	9.2 (5.6–14.7)		8	16.6 (5.9–38.5)
Cough	36	10.6 (6.5–16.9)		10	9 (3.7–19.9)
Muscle pain	12	5.2 (2.6–10.0)		3	1.6 (0.5–5.0)
Loss of smell or taste	4	2.3 (0.7–7.6)		4	2.7 (0.9–7.7)
Other signs/symptoms	5	0.7 (0.2–2.2)		3	2.4 (0.7–8.1)
Hospitalization	1	0.7 (0.001–5.0)		0	NA

*Number of participants who reported the symptom among those who tested positive by serology.

### Estimating SARS-CoV-2 Infection among Populations in the 5 Districts

According to seroprevalence rates from this survey, cumulative estimated versus reported SARS-CoV-2 infections per 100,000 population were 13,100 versus 158 for Blantyre, 9,400 versus 24 for Karonga, 6,100 versus 51 for Lilongwe, 4,100 versus 13 for Mangochi, and 12,100 versus 51 for Mzimba North (Table 4). Overall, using an adjusted seroprevalence rate, we estimated 486,771 infections in the 5 districts during April–December 2020, compared with the 4,319 reported rPCR-confirmed cases under the national surveillance program, an underestimation by a factor of 113. Our seroprevalence results show that an estimated 7,800/100,000 persons in the 5 districts sampled were infected with SARS-CoV-2 during April–December 8, 2020; national case-based surveillance data reported 69/100,000 persons for the same period.

**Table 4:** Estimated number of cases in the 5 districts from the survey compared with the cases reported to the national surveillance system by facilities in Malawi, December 2020

District	Population*	Total district case estimates/100,000 population	Total district case estimates			Reported cases	Reported cases/100,000 population	Estimation factor
			Lower bound	Middle estimate	Higher bound			
Blantyre	1,304,357	13,100	117,392	170,871	243,915	2,065	158	82.7
Karonga	380,608	9,400	27,784	35,777	46,434	91	24	393.2
Lilongwe	2,770,840	6,100	96,979	169,021	282,626	1,412	51	119.7
Mangochi	1,224,716	4,100	31,843	50,213	75,932	157	13	319.8
Mzimba North	560,129	12,100	48,731	67,776	92,981	594	106	114.1
Total	6,842,977	7,800	393,161	486,771	599,102	4,319	69	112.9

*Population estimates are projections from the Malawi National Statistical Office, 2018 Housing Census report.

## Discussion

Our survey results highlight several public health challenges and adds insights about SARS-CoV-2 infection and disease surveillance in Malawi and similar low-income settings. Results show SARS-COV-2 prevalence was very low at the time of the survey but much higher during preceding months. Most infections detected by either rPCR or ELISA were asymptomatic and all but 1 of the remaining cases was mild. Only 1 participant reported being hospitalized, a proportion similar to those from other reports. The survey identified several risk factors associated with positive serology, including being an HFS, living in an urban area, and having an immunosuppressive condition or diabetes (Table 2).

The huge discrepancy between SARS-CoV-2 infections estimated based on our survey and the official national count from case-based surveillance was previously documented in Malawi (*7*) and surrounding regions (*9*–*11*). The high proportion of asymptomatic infections and limited access to testing might explain the difference because asymptomatic persons are unlikely to seek testing and diagnostic capacity limited access to testing in Malawi to persons with signs and symptoms and travelers.

Two COVID-19 waves in Malawi have increased the proportion of exposed persons (Appendix). Widespread undetected and unmitigated transmission of SARS-CoV-2 presents an environment conducive for developing variants, undermining efforts to contain the COVID-19 pandemic (*12*). With variants emerging, enhanced support is needed to strengthen outbreak readiness and response among health systems in Africa; surveys and genomic surveillance should be prioritized and integrated into disease response, to inform surveillance and response decisions (*12*).

rPCR-confirmed SARS-CoV-2 infection prevalence during the survey period was similar to the low test positivity from national surveillance data in October (1.6%) and November (0.9%) of 2020. This finding suggests that, although routine health facility–based data might be indicative of the extent of symptomatic infections and disease trends in the community and case-based surveillance useful for monitoring trends in SARS-CoV-2 burden, these data might be insufficient for guiding public health actions to address the full extent of community transmission, driven in part by undiagnosed mild and asymptomatic infections. Alternative approaches, such as sentinel and syndromic surveillance, population-based surveys, and additional testing options, including rapid diagnostic tests or self-testing, are urgently needed to understand and respond to community transmission and prioritize and monitor effects from interventions, including vaccines.

The proportion of persons with asymptomatic SARS-CoV-2 infections in this survey is higher than in most previous studies, which have reported 35%–74% asymptomatic infections (*9*,*13*,*14*). Only 1 seropositive participant reported being hospitalized in the previous 6 months. The high proportion of young participants (median ages were 32 years among community participants and 35 years among HFS), reflective of the national age pyramid (*7*), might explain the predominance of asymptomatic or mild manifestations. In addition, fewer than one quarter of participants reported >1 underlying condition associated with an increased risk for severe disease, reflective of health conditions relative to the age distribution. Proportions of the population at risk for severe COVID-19 disease have been estimated at 16% in Africa and 31% in Europe but <4% in Malawi (*15*). The fact that most SARS-CoV-2 infections do not progress to symptomatic disease aligns with the low levels of illness and death from COVID-19 disease in Africa compared with Asia, Europe, and the Americas during the first wave (*16*).

The most critical public health outcomes of SARS-CoV-2 infection are severe disease and death, which in this survey were rare and have remained much lower in Africa than in Western nations after introduction and spread of Beta and Delta variants. Our findings highlight the need to identify context-specific predictors of severe disease and death, which would inform design of national response strategies proportionate to disease burden and public health resources.

The finding of higher prevalence of infection among HFS than the general population is consistent with findings from other studies (*17*,*18*). Because healthcare workforces in low-income countries are acutely limited, interventions and policies should prioritize efforts to maintain health services by protecting health workers including providing vaccinations and appropriate personal protective equipment. Higher prevalence among urban than rural participants in Malawi, consistent with findings from modeling studies in the region (*19*), was not unexpected because urban areas are more associated with overcrowding, indoor gatherings, and international travel (*20*). Based on testing numbers from each district, national case-based surveillance disease distribution data might have been influenced by testing volume and availability by district rather than reflecting the actual disease burdens by district observed in our results. Correcting unequal access to testing might balance statistical disease distribution patterns; conveying realistic perception of personal risk and the need to reduce associated risk reduction behaviors to the public and efforts to expand public health policy would also likely help address disparities.

Although diabetes has been associated with increased severity of COVID-19 manifestations (*21*) because of its effects on glucose homeostasis, inflammation, immune status, and activation of the renin-angiotensin-aldosterone system, little has been known about its effect on susceptibility to SARS-CoV-2 infection (*22*). This survey provides additional evidence on vulnerability of persons with diabetes to SARS-COV-2 infection. Reliance on self-reported diabetes status could be a limitation, but any misclassification would likely be nondifferential and only have biased the association toward equality.

Among other potential limitations, the Wantai ELISA test might have misclassified antibody status in a proportion of participants based on sensitivity and specificity limits (*23*). Our reliance on participant recall for some data, including presence of signs and symptoms in the 6 months before the survey and underlying health conditions, made data liable to recall bias. A higher proportion of HFS reported underlying conditions than community participants, which might be attributable to differences in health awareness. In addition, the target community participant sample size was not achieved. Refusal to participate in our survey by some communities introduced a small selection bias and also highlights factors such as distrust of health systems and misconceptions or disbelief related to SARS-CoV-2 that influence willingness to accept SARS-CoV-2 testing (*6*). Efforts to engage with communities to improve understanding and address misconceptions and other drivers of behavior should be incorporated into routine community messaging and strategies.

## Conclusion

Routine case-based surveillance might reflect trends in symptomatic disease prevalence but highly underestimate the full extent of community transmission. National COVID-19 response in low-income settings needs to use alternative surveillance and testing strategies to accurately track transmission and the effectiveness of interventions. Most infections recorded in this survey were asymptomatic, suggesting the need for research on predictors of symptomatic disease to inform development of contextualized and proportionate surveillance and response strategies.

Appendix Additional information about study of SARS-CoV-2 infection in Malawi, 2020.
