# Supplementary material for: SARS-CoV-2 Prevalence in Malawi Based on Data from Survey of Communities and Health Workers in 5 High-Burden Districts, October 2020
Source: Emerg Infect Dis. 2022 Dec;28(Suppl 1):S76–84. doi: 10.3201/eid2813.212348 (PMC9745213; doi:10.3201/eid2813.212348)
Supplement: Appendix — Additional information about study of SARS-CoV-2 infection in Malawi, 2020. [file 21-2348-Techapp-s1.pdf]

Article DOI: https://doi.org/10.3201/eid2813.212348

SARS-CoV-2 Prevalence in Malawi Based on Survey of Communities and Health Workers in 5 High-Burden Districts, October 2020

Appendix

Appendix Table 1. Total population and cumulative number of SARS-CoV-2 infections per 100,000 population in Malawi and in the 5 districts

District	NSO 2020 population projection	Cumulative confirmed SARS-CoV-2 infections (as of 2020 Oct 14) *	Cumulative no. confirmed infections per 100,000
Blantyre	1,304,357	1,976	151
Lilongwe	2,770,840	1,353	49
Karonga	380,608	82	22
Mangochi	1,224,716	149	12
Mzimba North	560,129	566	101
National total	18,449,828	5,829	30

Extracted from Malawi Daily COVID-19 Situation Report for October 14, 2020
*NSO, National Statistics Office

Appendix Table 2. Details of the number of health facilities sampled by district and urban/rural stratification and target sample size per facility

	Urban		Rural		
District	No. facilities	No. participants per facility	No. facilities	Participants per facility	Total participants
Blantyre	5	68	3	20	400
Lilongwe	5	68	3	20	400
Mzuzu	5	68	3	20	400
Mangochi	1*	60	7	20	200
Karonga	1*	60	7	20	200
Total	17	1,140	23	460	1,600

* Karonga and Mangochi included health facility staff from the district hospital

Appendix Table 3. Health facility staff cadres and weighted proportions

Health facility staff cadre	No.	Weighted seroprevalence (95% CI)
Nurses	216	9.5 (3.1–25.1)
Doctor	9	3.9 (0.02–90.5)
Clinical officer	45	32.8 (9.9–68.5)
Medical assistant	23	0.8 (0.10–8.6)
Environmental health officers	9	3.2 (0.03–77.5)
Health surveillance assistants, including community health worker and mobilizers	179	7.3 (3.8–13.6)
Laboratory staff	29	16.5 (4.7–44.1)
Pharmacists	6	28.1 (0.10–99.4)
Pharmacy technicians	10	7.2 (0.2–72.3)
Data entry staff	39	8.2 (2.1–27.1)
Cleaner	154	3.5 (1.4–8.6)
Hospital attendant/case care worker or patient attendant	93	12.3 (6.2–22.7)
Expert HIV client volunteer	29	32.6 (12.3–62.4)
HIV testers/counselors (includes HIV diagnostic assistants)	51	4.6 (1.1–17.6)
Security officer/guard/grounds labor/maintenance	45	1.6 (0.3–8.7)
Other	63	9.6 (2.3–31.9)
